# Surgical treatment of preauricular fistulas: a 12-year single-center clinical observation

**DOI:** 10.1186/s12893-023-02198-x

**Published:** 2023-09-30

**Authors:** Ke Li, Yan Hao, Jiuzhou Zhao, Leping Zhou, Yongyan Wu, Xianhai Zeng, Wei Gao, Xiangmin Zhang

**Affiliations:** 1 Department of Otolaryngology, Shenzhen Key Laboratory of E.N.T, Institute of E.N.T Shenzhen, Longgang E.N.T Hospital, Guangdong Province, People’s Republic of China; 2 Department of Otolaryngology, Shenzhen Hospital, Southern Medical University, Guangdong Province, People’s Republic of China

**Keywords:** Preauricular fistula, Surgery, Fistula resection, Operative duration, Recurrence

## Abstract

**Objective:**

This study aimed to assess the effects of surgical timing and approach on operative duration, postoperative suture removal time, and postoperative recurrence rate in the management of preauricular fistula. A 12-year single-center clinical observation was conducted to analyze the potential effects of different surgical strategies on these critical outcomes.

**Methods:**

The clinical data from 576 (782 ears) patients who underwent surgical resection for preauricular fistulas were examined in this retrospective study. The patients were classified into various groups based on differences in operative duration, surgical techniques and the use of intraoperative magnifying equipment. Furthermore, the specific data on operative duration, postoperative suture removal time, and postoperative recurrence rate were also recorded.

**Results:**

The average operative duration for 782 ears and the average time required for postoperative suture removal were determined to be (34.57 ± 4.25) min and (3.62 ± 0.76) days, respectively. Among the cases examined, recurrence occurred in 13 ears, but all of them were cured after a second surgery, resulting in a recurrence rate of 1.67% (13/782). Interestingly, the operative and postoperative suture removal time was prolonged during the infection period (*P* < 0.05). The postoperative recurrence rate was significantly higher in the absence of magnifying equipment, as compared to those with the use of a microscope with 2.5× magnification (*P* < 0.05). No statistically significant differences were noted in the recurrence rate when comparing different anesthesia methods and types of surgical incisions, as well as the intraoperative use of methylene blue, and partial removal of cartilage of the pedicle (*P* > 0.05).

**Conclusion:**

The use of methylene blue, partial removal of the cartilage of the pedicle, and surgical incision during preauricular fistula resection did not affect the operative duration, postoperative suture removal time, and postoperative recurrence rate. Therefore, surgeons can select their preferred approaches based on their individual practices and patient-specific situations. However, the use of magnifying equipment during surgery is associated with a reduced risk of recurrence.

## Introduction

Congenital preauricular fistula refers to a congenital malformation observed in the field of otolaryngology. The most prominent clinical manifestation in affected individuals is a congenital malformation in the external ear. This condition exhibits regional and racial variations in prevalence, with the highest incidence in Africa. Notably, the incidence of preauricular fistulas in China exceeds that in developed regions such as Europe and the United States [1, 2]. Preauricular fistulas commonly do not cause any noticeable symptoms. However, some patients may present with localized manifestations, such as erythema, swelling, pain, pus discharge, swelling of adjacent soft tissues, and skin deterioration in the presence of an infection. These symptoms can seriously affect facial hygiene and overall quality of life [3, 4].

Surgery remains the most effective treatment method for complex preauricular fistulas that are susceptible to infection and pose challenges in complete removal during the surgery, resulting in a high postoperative recurrence rate [5, 6]. The recurrence after preauricular fistula resection is currently a considerable clinical concern. Addressing this concern requires the identification of an effective method to reduce the risk of recurrence by selecting appropriate surgical techniques [7, 8]. Therefore, to enhance our clinical knowledge and contribute to strategies for reducing recurrence, we assessed the effects of the surgical timing and approach for preauricular fistula on the operative duration, postoperative suture removal time, and postoperative recurrence rate. This assessment was carried out through the observation of preauricular fistula surgeries in a single medical center for 12 years.

## Materials and methods

### Ethics statement

The research protocol involving clinical samples was approved by the Ethical Committee of Longgang ENT Hospital (Shenzhen, China; NO. 2022-0032), which was conducted in accordance with the established guidelines of the *Declaration of Helsinki*. Prior to participation, all human subjects and the parents or legal guardians of minor participants were provided with detailed information regarding the study, its purpose, and the collection of anonymous specimens. Informed consent was obtained from all individuals involved in the study.

### Inclusion criteria of patients

The inclusion criteria were listed as follows: (i) patients who had not undergone surgical treatment for preauricular fistula prior to their initial visit to our hospital; (ii) patients who did not show any indication of healing after local incision of the fistula; (iii) patients who had comprehensive clinical records and complete follow-up data.

### Exclusion criteria of patients

The criteria for excluding patients from the study were described below: (i) patients who showed evidence of healing after local incision of the fistula; (ii) patients who experienced relapse after previous surgical intervention; (iii) patients who were affected by concurrent skin disorders in the vicinity of the fistula site; (iv) patients who were diagnosed with cardiovascular diseases; (v) patients who had severe organ diseases, immunodeficiency, and other critical medical conditions.

### Clinical data

This retrospective study involved the analysis of clinical data from 576 patients (782 ears in total) who underwent preauricular fistula resection at Longgang ENT Hospital (Shenzhen, China). The study spanned a period of 12 years, from November 2009 to November 2021. The patient cohort consisted of 239 males and 337 females, with ages ranging from 10 months to 78 years and a median age of 22.5 ± 9.50 years (Table 1).

**Table 1:** General clinical data of the patients (n = 576 patients, 782 ears)

Variable	Number of patients, n (%)
**Age, years**	
1–9	202 (35.07)
10–19	113 (19.62)
20–29	110 (19.10)
30–39	83 (14.41)
40–49	39 (6.77)
50–59	21 (3.65)
60–69	6 (1.04)
70–79	2 (0.3)
**Sex**	
Male	239 (41.49)
Female	337 (58.51)
**Preauricular fistula**	
Unilateral	370 (64.24)
Bilateral	206 (35.76)
**Presence of infection**	
Yes	623 (79.67)
No	159 (20.33)

### Selection of surgical timing and approach

The selection of the optimal timing and approach for surgery was determined based on the patient’s condition at different stages. Patients were categorized into three groups: those who underwent surgical treatment during the active infection phase, those who received surgery after successful control of infection through incision and drainage of pus, and those who did not present any signs of infection requiring surgical intervention.

### Surgery methods

**Fig. 1:**
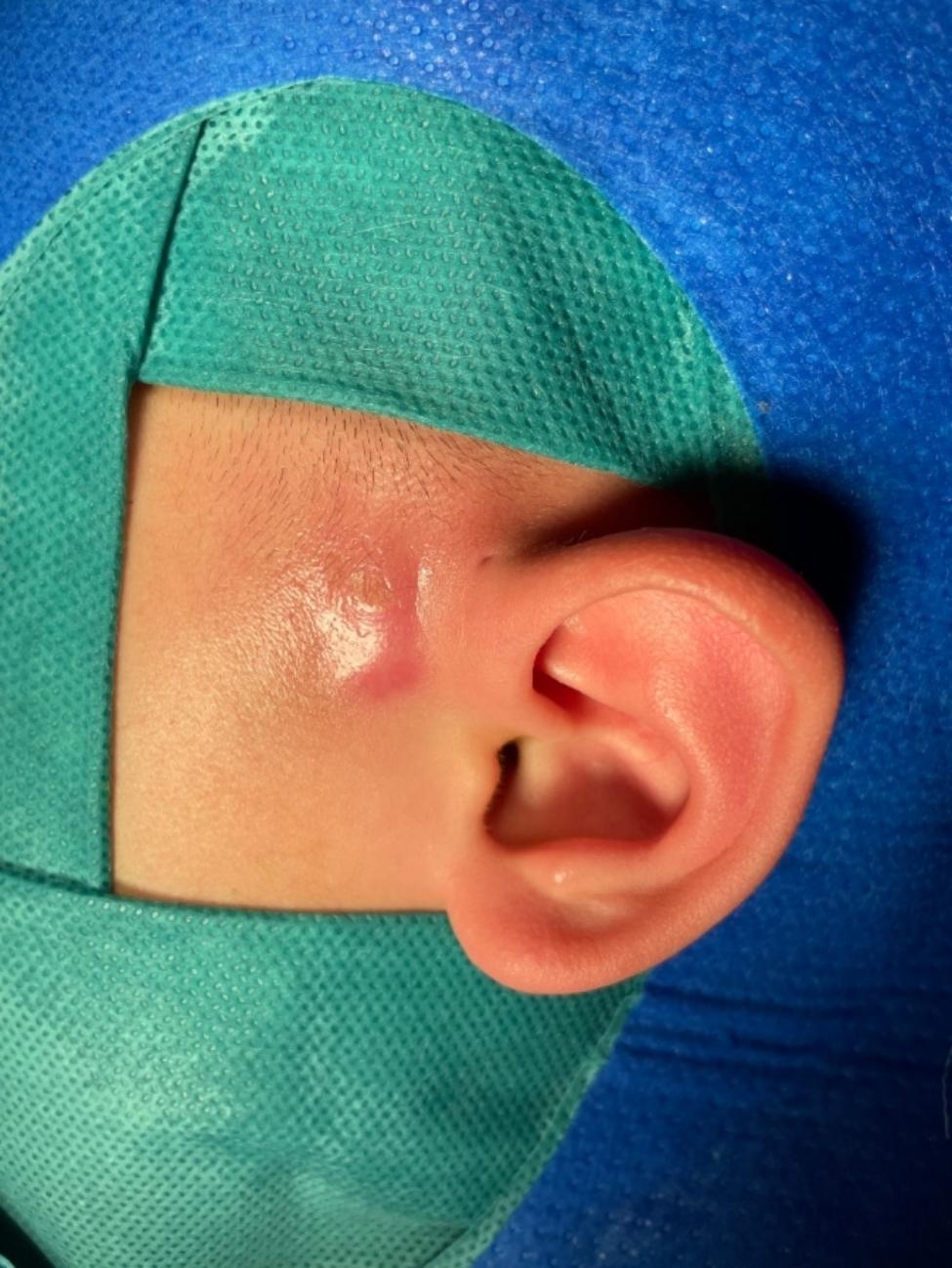
Location of representative preauricular fistula infection. Note: The figure illustrates an infected preauricular fistula positioned anterior to the tragus region of the ear.

**Fig. 2:**
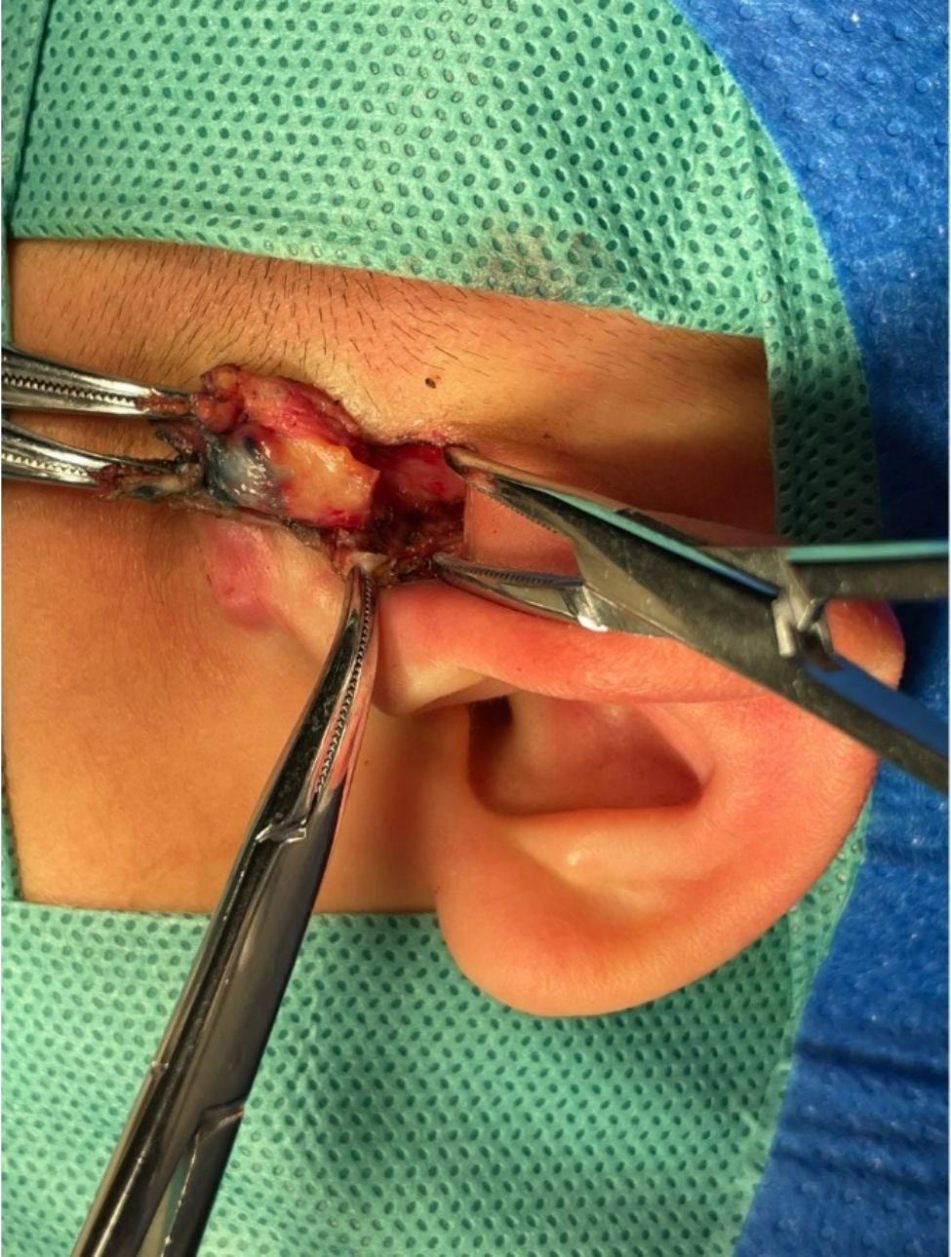
Surgery for preauricular fistula removal with methylene blue injection. Note: After the administration of methylene blue, a shuttle incision is made around the fistula, followed by the removal of the fistula.

**Fig. 3:**
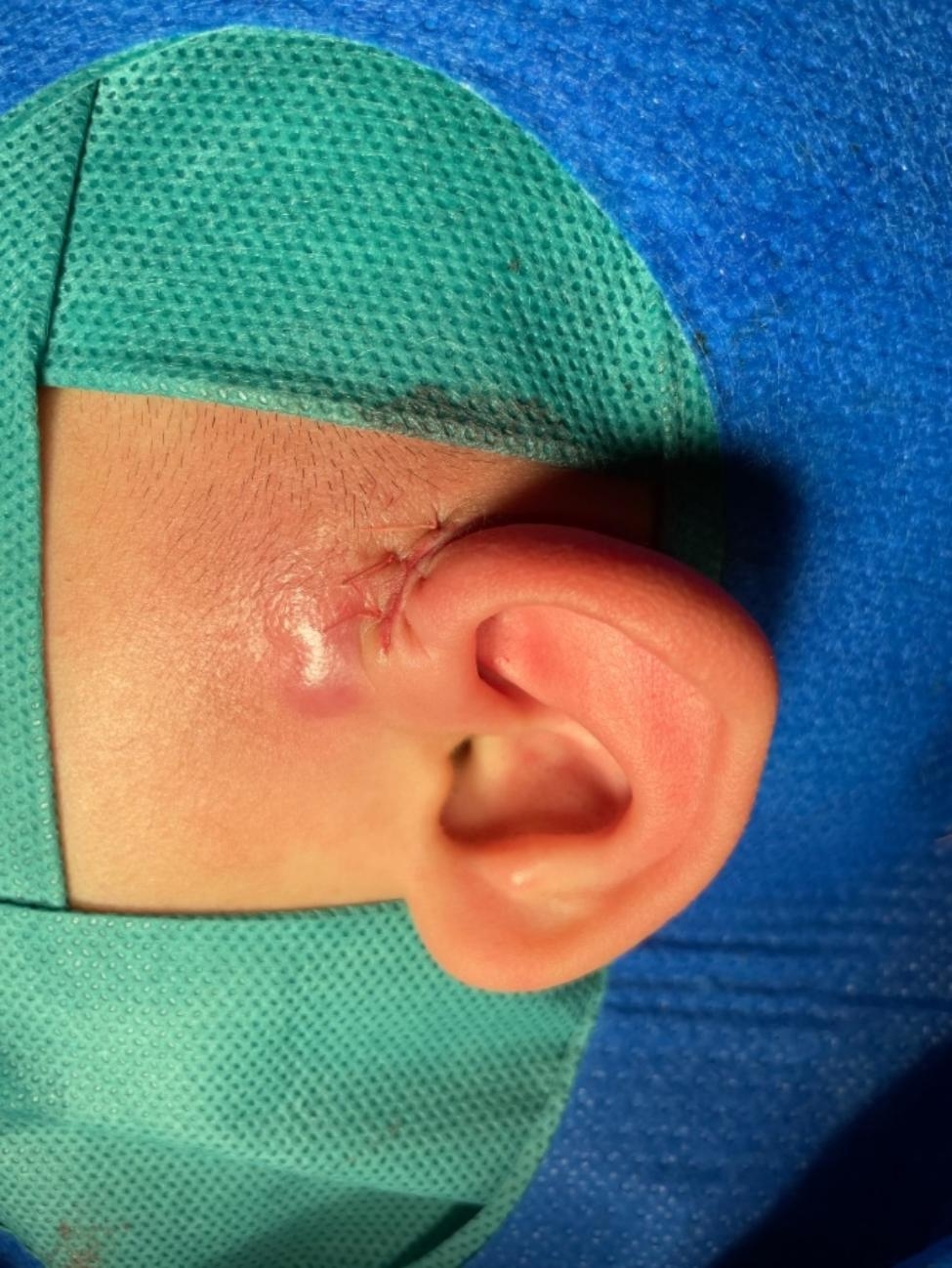
Postoperative outcome after complete excision of preauricular fistula. Note: This figure demonstrates the condition after complete excision of the preauricular fistula through surgery.

**Fig. 4:**
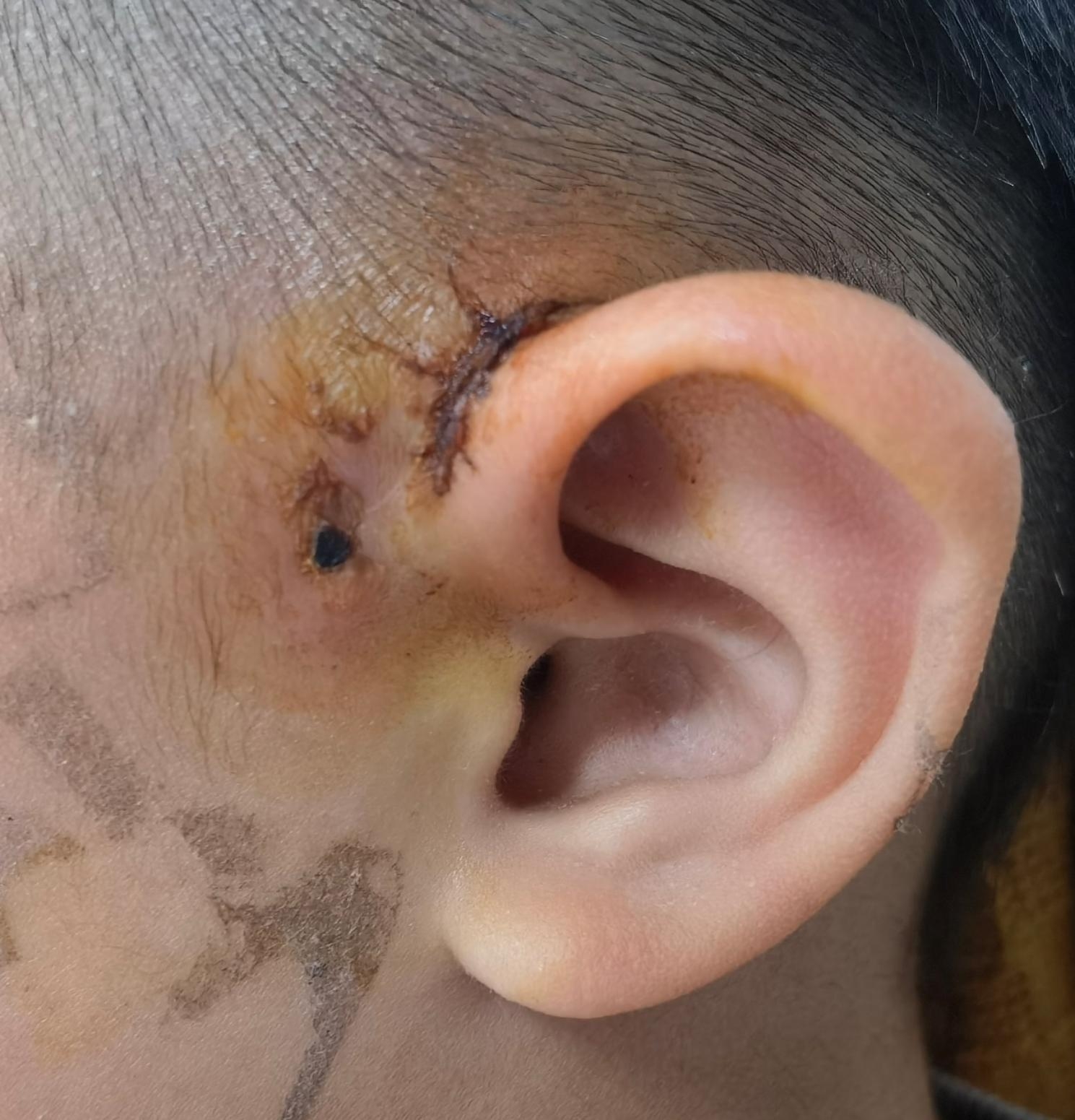
Early postoperative evaluation. Note: This figure depicts the evaluation conducted one week after the surgical resection of the preauricular fistula resection, providing insights into the immediate postoperative period.

**Fig. 5:**
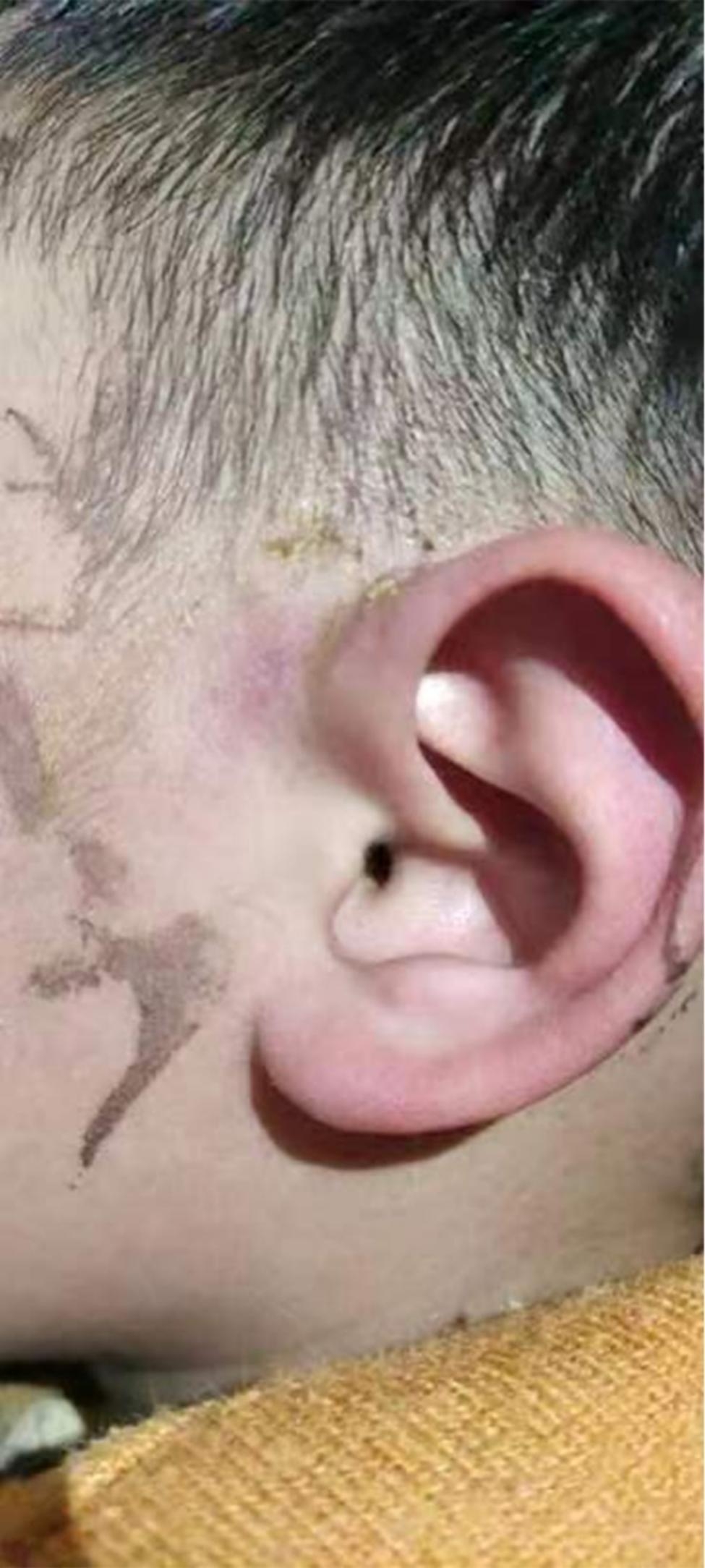
Postoperative evaluation after a longer period of time. Note: The figure displays the status of the patient one month after preauricular fistula resection, offering an assessment of the extended recovery process.

Prior to surgery, all patients received either general or local anesthesia and underwent the surgical intervention performed in a supine position, with the affected ear positioned in an upward direction. As part of the preoperative assessment, a lacrimal probe was inserted through the fistula opening to carefully explore the trajectory and extent of the sinus tract. Additionally, in some patients, a small amount of methylene blue dye was injected through the fistula opening for enhanced visualization and evaluation. The fistula was separated from the cartilage attachment of the pedicle of the ear. In cases where the surgical field was obstructed by a segment of the pedicle cartilage, that segment was excised. Additionally, during surgeries performed during the active infection phase, a scraper was used to remove the necrotic and granulation tissues from the pus-filled cavity. During the intraoperative phase, precautionary measures were implemented to prevent damage to the superficial temporal artery. In cases of accidental injury, hemostasis was promptly achieved by ligating the major blood vessels to arrest bleeding, while bipolar electrocoagulation was employed for the remaining smaller vessels. Subsequently, the incision was thoroughly cleansed and irrigated before being carefully sutured. For patients with more extensive partial defects after resection, a provisional flap repair technique was required to address the specific needs (Figs. 1, 2, 3, 4 and 5).

### Observation of indicators

All enrolled patients were followed up for a period from 3 months to 12 years. Upon completion of the follow-up period, crucial variables, such as operative duration, postoperative suture removal time, and postoperative recurrence rate, were systematically recorded and analyzed for patients who underwent surgery with different surgical timing and approaches.

### Statistical analysis

SPSS 25.0 (IBM, Armonk, NY, USA) was used for data analysis in this study. The measurement data were described as mean ± standard deviation and analyzed using the *t*-test. The count data were presented as the number of cases or percentages (%) nd evaluated using the chi-square test. The significance level for all tests was set at α = 0.05. *P* < 0.05 was considered statistically significant.

## Results

### Demographic and surgical characteristics of the patients

Among the total 782 ears (576 patients) included in this study, 202 ears underwent surgery during the infection phase. Additionally, 421 ears were operated on after achieving proper control of infection through incision and drainage of pus. Moreover, 159 ears received surgical treatment during the non-infection period. In terms of anesthesia usage, 561 ears were surgically managed under general anesthesia and 15 under local anesthesia. Regarding the use of equipment, 237 ears were subjected to surgical procedures under a microscope, while 264 ears were operated on under the assistance of 2.5× magnification device. Furthermore, 281 ears were operated on without the use of any magnifying equipment. In terms of the administration of methylene blue, 553 ears received the dye, and 229 ears did not. The cartilage segment of the pedicle of the ear was removed in 306 ears, whereas it was left intact in 476 ears. Regarding the incision technique, a single shuttle incision was made in 475 ears, a double incision was made in 228 ears, and a skin flap was made in 79 ears (Table 2).

**Table 2:** General information of surgery (n = 576 patients, 782 ears)

Variable	Number of patients, n (%)
**Timing of surgery**	
During infection phase	202 (25.83)
After controlling the infection	421 (53.84)
During the non-infection phase	159 (20.33)
**Anesthesia method**	
General anesthesia	561 (97.40)
Local anesthesia	15 (2.60)
**Use of magnifying equipment**	
Microscope	237 (30.31)
2.5× magnifying glass	264 (33.76)
No equipment used	281 (35.93)
**Use of methylene blue**	
Yes	553 (70.72)
No	229 (29.28)
**Parital removal of the cartilage of the pedicle**	
Yes	306 (39.13)
No	476 (60.87)
**Incision type**	
Single shuttle incision	475 (60.74)
Double incision	228 (29.16)
Skin flap	79 (10.10)

### Average operative duration, postoperative suture removal time, and postoperative recurrence rate

The average operative duration for 782 ears was (34.57 ± 4.25) min, and the average postoperative suture removal time was (3.62 ± 0.76) days. Among the cohort, 13 ears had a recurrence, but were cured after the second surgery. This finding corresponds to an occurrence rate of 1.67% (13/782).

### Operative duration and postoperative suture removal time

The operative and postoperative suture removal time was extended when surgery was performed during the infection phase compared with that performed during the non-infection phase (*P* < 0.05). No statistically significant difference was noted in the operative duration and postoperative suture removal time when evaluating different anesthesia methods, types of surgical incisions, intraoperative use of methylene blue, and partial removal of the cartilage of the pedicle (*P* > 0.05) (Table 3).

**Table 3:** Comparison of operative duration and postoperative suture removal time

Variable	Operative duration (min)	Postoperative suture removal time (d)
**Timing of surgery**		
During infection phase	37.90 ± 5.19	3.90 ± 1.03
After controlling the infection	34.20 ± 3.55	3.70 ± 0.82
During the non-infection phase	31.10 ± 2.29	3.03 ± 0.44
*t*	18.855	15.678
*P*	0.000	0.000
**Use of magnifying equipment**		
Microscope	34.21 ± 4.04	3.66 ± 0.80
2.5× magnifying glass	36.70 ± 4.82	3.64 ± 0.73
No equipment used	36.83 ± 5.39	3.60 ± 0.69
*t*	0.700	0.172
*P*	0.485	0.678
**Anesthesia method**		
General anesthesia	34.49 ± 4.28	3.60 ± 0.70
Local anesthesia	34.59 ± 4.50	3.64 ± 0.77
*t*	0.089	0.218
*P*	0.929	0.828
**Use of methylene blue**		
Yes	34.45 ± 4.12	3.64 ± 0.80
No	34.58 ± 4.60	3.60 ± 0.74
*t*	0.388	0.650
*P*	0.698	0.516
**Parital removal of the cartilage of the pedicle**		
Yes	34.33 ± 4.09	3.63 ± 0.79
No	34.70 ± 4.54	3.61 ± 0.73
*t*	1.156	0.362
*P*	0.248	0.718
**Incision type**		
Single shuttle incision	34.05 ± 4.10	3.59 ± 0.69
Double incision	34.40 ± 4.43	3.66 ± 0.75
Skin flap	34.80 ± 4.52	3.70 ± 0.78
*t*	2.092	2.985
*P*	0.148	0.084

### Univariate analysis of postoperative recurrence

All patients were followed up for 3 months to 12 years (average follow-up time: 68.3 months). The postoperative recurrence rate was significantly higher in cases where magnifying equipment was not utilized, as compared to surgeries performed with the use of a microscope or 2.5× magnification (*P* < 0.05). On the other hand, there were no statistically significant differences in recurrence rates when comparing surgeries performed during infection and non-infection phases (*P* > 0.05) (Table 4). The findings underscore the vital role of magnifying equipment in optimizing surgical outcomes.

**Table 4:** Univariate analysis of postoperative recurrence n (%) (n = 576 patients, 782 ears)

Variable	Postoperative recurrence	No postoperative recurrence	χ2	*p*
**Timing of surgery**				
During infection phase	5 (2.48)	197 (97.52)	3.650	0.161
After controlling the infection	8 (1.90)	413 (98.10)		
During the non-infection phase	0(0.00)	159 (100.00)		
**Use of magnifying equipment**				
Microscope	0 (0.00)	237 (100.00)	14.047	0.001
2.5× magnifying glass	2(0.76)	262 (99.24)		
No equipment used	11(3.91)	270(96.09)		
**Anesthesia method**				
General anesthesia	12 (2.14)	549 (97.86)	0.328	0.567
Local anesthesia	0 (0.00)	15 (100.00)		
**Use of methylene blue**				
Yes	8 (1.45)	545 (98.55)	0.537	0.464
No	5(2.18)	224 (97.82)		
**Parital removal of the cartilage of the pedicle**				
Yes	4(1.31)	302 (98.69)	0.388	0.534
No	9 (1.89)	467 (98.11)		
**Incision type**				
Single shuttle incision	9 (1.89)	466 (98.11)	0.400	0.819
Double incision	3 (1.32)	225 (98.68)		
Skin flap	1(1.27)	78(98.73)		

## Discussion

In the present study, we assessed the effects of the surgical timing and approach on the operative duration, postoperative suture removal time, and postoperative recurrence rate in cases of preauricular fistula. It was observed that conducting surgery during the infection phase was associated with a significant increase in both operative duration and postoperative suture removal time. However, the timing of surgery did not exert any notable effect on the recurrence rate. The use of magnifying equipment during surgery has been found to affect the postoperative recurrence rate. Conversely, the use of methylene blue, partial removal of the cartilage of the pedicle, and specific surgical incisions during preauricular fistula resection did not demonstrate any significant effects on the operative duration, postoperative suture removal time, and postoperative recurrence rate. Thus, the surgeons can exercise their professional judgment and select the most suitable approach based on individual preferences and patient-specific conditions.

Patients with congenital preauricular fistula manifest squamous epithelial cells in their fistulas, which are abundant in sebaceous and sweat glands, as well as hair follicles. In cases where the condition of the fistula deteriorates, the abovementioned constituents of the squamous epithelial cells are prone to shedding and accumulating in the cavity. This condition, combined with the presence of additional branches in the fistula, contributes to impaired outflow, thus rendering the fistula highly susceptible to infection [9, 10]. The occurrence of infection triggers the formation of inflammatory granulation tissues and localized scarring. Thus, when surgery is performed during the infection phase, additional time is required for the complete removal of these inflammatory granulation tissues and scars to mitigate the risk of local recurrence. Simultaneously, surgical bleeding intensifies, resulting in visual obscuration in the surgical field and hampers the pace of the surgery, thus extending the overall operative duration [11, 12]. Moreover, the removal of inflammatory granulation tissue and scars is accompanied by an increase in damage to surrounding tissues. Conversely, the compromised visibility in the surgical field requires the enlargement of the incision to achieve improved visualization during the separation of the fistula and elimination of the inflammatory granulation tissue. Consequently, these factors contribute to delayed postoperative recovery and prolonged duration for suture removal [13].

The recurrence of preauricular fistulas primarily arises from the incomplete removal of the fistula, as well as the surrounding scars and inflammatory granulation tissue. Hence, it is recommended that during surgical intervention, whether conducted during periods of infection or non-infection, utmost care should be taken to completely remove the inflammatory granulation tissue. By adopting this approach, the recurrence rate remains consistent, independent of the timing of surgery, aligning with the research findings of Wang et al. [14]. Congenital preauricular fistulas are characterized by more complex branches. In conventional surgical practices, the identification and management of the fistula and associated lesioned tissues are primarily accomplished by visually marking these tissues following the application of methylene blue staining. However, the accumulation of cheese-like obstructive material and inflammatory granulation tissue in the fistula poses challenges to the staining process. Consequently, methylene blue often fails to penetrate and reach the distal branches of the fistula [15–17]. Therefore, solely relying on visual inspection without aids proves inadequate in accurately identifying the presence of the fistula, thereby leaving behind a greater quantity of inflammatory granulation tissue and increasing the risk of postoperative recurrence [18, 19]. The application of microscopic or magnifying equipment, particularly microscopes, allows for enhanced clarity in distinguishing inflammatory granulation tissues, scars, and residual fistulas. Therefore, incorporating such equipment into the surgical procedure facilitates the complete removal of these tissues while minimizing damage to small vessels and healthy surrounding tissues, whereby mitigating the risk of postoperative infection and recurrence [20–22]. Despite these precautionary measures, there remains a potential for recurrent postoperative infections, which can prolong the recovery process and considerably amplify the pain and challenges associated with treatment for the affected patients.

Nonetheless, there are a few limitations to our study. Firstly, the retrospective design introduces the potential for selection bias, as the participants may not have been selected in a completely randomized manner. Secondly, the limited number of reported cases and the relatively short follow-up duration, with the longest period being less than 12 years, pose challenges in accurately determining the highest postoperative recurrence rate. This uncertainty might affect the reliability of the assessment. To further validate and strengthen our findings, future investigations should prioritize studies with larger sample sizes and extended follow-up periods or consider incorporating multicenter clinical randomized controlled trials.

## Conclusion

The use of methylene blue, partial removal of the cartilage of the pedicle, and surgical incision during preauricular fistula resection did not impart a significant impact on the operative duration, postoperative suture removal time, and postoperative recurrence rate. Therefore, the surgeons can select their preferred approach based on their individual practices and the specific circumstances of each patient. Notably, the use of magnifying equipment during surgery has demonstrated the potential to diminish the recurrence rate, underscoring its clinical utility as an adjunctive tool to enhance surgical outcomes.

## Data Availability

Datasets are available on request from the corresponding author on reasonable request. The raw data and all related documents supporting the conclusions of this manuscript will be made available by the authors, without undue reservation, to any qualified researcher.
